# Did we learn something positive out of the COVID-19 pandemic? Post-traumatic growth and mental health in the general population

**DOI:** 10.1192/j.eurpsy.2021.2263

**Published:** 2022-01-10

**Authors:** Giulia Menculini, Umberto Albert, Valeria Bianchini, Claudia Carmassi, Giuseppe Carrà, Francesca Cirulli, Bernardo Dell’Osso, Michele Fabrazzo, Francesco Perris, Gaia Sampogna, Maria Giulia Nanni, Maurizio Pompili, Gabriele Sani, Umberto Volpe, Alfonso Tortorella

**Affiliations:** 1 Department of Psychiatry, University of Perugia, Perugia, Italy; 2 Department of Medicine, Surgery and Health Sciences, University of Trieste, Trieste, Italy; 3 Department of Mental Health, Psychiatric Clinic, Azienda Sanitaria Universitaria Giuliano-Isontina—ASUGI, Trieste, Italy; 4 Department of Life, Health and Environmental Sciences, Psychiatric Unit: Trattamenti Riabilitativi Psicosociali, Interventi Precoci, TRIP, Psychosocial Rehabilitation Treatment, Early Interventions University Unit, University of L’Aquila, L’Aquila, Italy; 5 Department of Clinical and Experimental Medicine, University of Pisa, Pisa, Italy; 6 Department of Medicine and Surgery, University of Milan-Bicocca, Milan, Italy; 7 Center for Behavioral Sciences and Mental Health, National Institute of Health, Rome, Italy; 8 Department of Mental Health, University of Milan, Milan, Italy; 9 Department of Biomedical and Clinical Sciences “Luigi Sacco”, University of Milan, Milan, Italy; 10 Department of Health Sciences, Aldo Ravelli Center for Neurotechnology and Brain Therapeutic, University of Milan, Milan, Italy; 11 Department of Psychiatry, University of Campania “Luigi Vanvitelli”, Largo Madonna delle Grazie, Naples, Italy; 12 Department of Biomedical and Specialty Surgical Sciences, Institute of Psychiatry, University of Ferrara, Ferrara, Italy; 13 Department of Neurosciences, Mental Health and Sensory Organs, Faculty of Medicine and Psychology, Sapienza University of Rome, Rome, Italy; 14 Department of Neuroscience, Section of Psychiatry, Università Cattolica del Sacro Cuore, Rome, Italy; 15 Department of Psychiatry, Fondazione Policlinico Agostino Gemelli IRCCS, Rome, Italy; 16 Clinical Psychiatry Unit, Department of Clinical Neurosciences, Università Politecnica delle Marche, Ancona, Italy

**Keywords:** pandemic, trauma, post-traumatic growth, resilience, mental health

## Abstract

**Background:**

When facing a traumatic event, some people may experience positive changes, defined as posttraumatic growth (PTG).

**Methods:**

Understanding the possible positive consequences of the pandemic on the individual level is crucial for the development of supportive psychosocial interventions. The present paper aims to: 1) evaluate the levels of PTG in the general population; 2) to identify predictors of each dimension of post-traumatic growth.

**Results:**

The majority of the sample (67%, *N* = 13,889) did not report any significant improvement in any domain of PTG. Participants reported the highest levels of growth in the dimension of “appreciation of life” (2.3 ± 1.4), while the lowest level was found in the “spiritual change” (1.2 ± 1.2). Female participants reported a slightly higher level of PTG in areas of personal strength (*p* < .002) and appreciation for life (*p* < .007) compared to male participants, while no significant association was found with age. At the multivariate regression models, weighted for the propensity score, only the initial week of lockdown (between 9-15 April) had a negative impact on the dimension of “relating to others” (*B* = −.107, 95% CI = −.181 to −.032, *p* < .005), while over time no other effects were found. The duration of exposure to lockdown measures did not influence the other dimensions of PTG.

**Conclusions:**

The assessment of the levels of PTG is of great importance for the development of ad hoc supportive psychosocial interventions. From a public health perspective, the identification of protective factors is crucial for developing ad-hoc tailored interventions and for preventing the development of full-blown mental disorders in large scale.

## Introduction

The COVID-19 pandemic has had a profound negative impact on the mental health of the general population [1–3]. The pandemic can be considered as a new type of traumatic stressor, being an unexpected event, affecting the whole population worldwide and causing a severe disruption of daily routine life [4–6]. Recent research suggests that traumatic stress reactions, including intrusive reexperiencing and heightened arousal, are frequent during the pandemic [7] and may be due to its direct threats to important life resources of the general population, such as safety, health, income [8], work, housing, and social support [9,10]. Furthermore, the traumatic stress reactions to the COVID-19 pandemic may be worsened by the indirect exposure to the pandemic, for example, via mass-media coverage and the phenomenon of infodemic [11,12]; by the psychosocial consequences of the pandemic, in terms of unemployment, isolation [13], nonsudden illness/death [14–16]; and by the lack of clear and reliable therapeutic guidelines for the management of the COVID-19 infection [17].

The negative consequences of the pandemic on the mental health may vary in different target populations, such as healthcare professionals, people infected by the COVID-19, people living with disabilities or affected by chronic physical and mental disorders [18] or special population, such as pregnant women [19–24], elderly [25,26] or young people [27–30]. In particular, the psychiatric and psychological consequences of the pandemic on the general population mainly include high levels of distress [31,32] and of post-traumatic reactions [33–35], social isolation with suicidal ideation [36–39], depressive and anxiety symptoms and sleep disorders [40–44]. A high prevalence of mental exhaustion, burn-out syndrome and insomnia has been found in healthcare workers [45–47]. In disabled people and in those with pre-existing mental health problems, an increased risk of treatment interruption of long-term treatments has been found, associated with relapses or symptoms worsening, as well as with a higher risk of being infected by the COVID-19 [48–53]. Specific risk factors identified for the development of these mental health disturbances include female gender, having previous psychiatric or physical disorders, loneliness, time spent on the Internet, and unemployment [54,55].

Although these different populations are exposed to the same traumatic event (i.e., the pandemic), its perception is highly variable, because it is mediated by individual psychological and social factors, such as coping strategies and resilience styles [56–60].

When facing a traumatic event, some people may also experience positive changes, the so-called posttraumatic growth (PTG) [61,62]. The PTG is a substantive, positive change in a person’s self-perceptions, relationships with others, and/or their personal philosophy of life, resulting after a traumatic experience [63,64]. PTG consists of five dimensions [65]: (a) changes in how people relate with others (i.e., an increased willing to express emotions or even accepting more likely help from others); (b) recognition of new possibilities (i.e., seen as an increased attitude to take new paths in life and redefine priorities); (c) a sense of greater personal strength (i.e., improved sense of self-efficacy, strength, and self-confidence); (d) changes toward spirituality (i.e., religious beliefs, spiritual matters, and existential/philosophical questions); and (e) greater appreciation of life (i.e., considering meaningful and worth in life’s little things).

Some studies [66–70] highlighted how a collective experience of trauma can help people reflecting on their traumatic experiences, as it would be the case for the COVID-19 pandemic [71]. Understanding the possible positive consequences of the pandemic on the individual level is crucial for the development of preventive and supportive psychosocial interventions for the general population [72–76]. Furthermore, the sociodemographic and clinical factors facilitating the positive adaptation to trauma may be worth to identify.

During the initial phase of the pandemic, Italy has been among the most severely hit countries, with high rates of COVID-related morbidity and mortality, high occupancy rate in intensive care units and extreme burden on the national health systems. Therefore, the Italian government issued severe public health measures, with lockdown and quarantine in order to limit the spread of the disease. The COvid Mental hEalth Trial (COMET) study is a multicentric, collaborative, notfunded trial carried out during the initial phase of the COVID-19 pandemic, targeting the Italian general population during the first wave of the lockdown [54,77].

Based on the COMET study, the present paper aims to: (a) evaluate the levels of PTG in a sample of the general population and (b) to identify predictors of each dimension of post-traumatic growth.

## Materials and Methods

The present paper is based on data collected in the COMET [54,77].

The COMET study has been coordinated by the University of Campania “Luigi Vanvitelli” (Naples), and includes other Italian university sites (Università Politecnica delle Marche [Ancona], University of Ferrara, University of Milan Bicocca, University of Milan “Statale,” University of Perugia, University of Pisa, Sapienza University of Rome, “Catholic” University of Rome, and University of Trieste) with the Center for Behavioral Sciences and Mental Health of the National Institute of Health in Rome. The COMET trial has been designed as cross-sectional study, adopting a snowball sampling procedure [77].

The main outcome measure considered in the present study is represented by the levels of Post Traumatic Growth, which have been evaluated by using the short form of the Post-Traumatic Growth Inventory (PTGI) [78]. The PTGI consists of 10 items, rated on a 6-point Likert scale (i.e., 0 = “I did not experience this change as a result of my crisis”; 5 = “I experienced this change to a very great degree as a result of my crisis”). Items are grouped in following five dimensions: (a) relating to others; (b) new possibilities; (c) personal strengths; (d) spiritual change; and (e) appreciation of life. It is calculated a total score, so that higher scores indicate higher levels of post-traumatic growth. Responses on the items were averaged to form the scale score, and the attainment of substantial PTG was indicated by an average score of 4 [79].

The survey includes also the following validated self-reported questionnaires: DASS-21 [80]; General Health Questionnaire—12 items version (GHQ) [81]; Obsessive–Compulsive Inventory—Revised version (OCI-R) [82]; Insomnia Severity Index (ISI) [83]; Suicidal Ideation Attributes Scale (SIDAS) [84]; Severity of Acute Stress Symptoms Adult Scale (SASS) [85]; the Impact of Event Scale—short version (IES) [86]; the UCLA loneliness scale—short version [87]; the Brief-COPE [88]; the Connor–Resilience Scale [89]; and the Multidimensional Scale of Perceived Social Support (MSPPS) [90]. Moreover, sociodemographic information (i.e., gender, age, civil status, level of education, number of cohabitations, geographical region, living in one of the most severely impacted area, working condition, and housing condition) have been collected through an ad hoc schedule.

This study is being conducted in accordance with globally accepted standards of good practice, in agreement with the Declaration of Helsinki and with local regulations.

Written informed consents have been collected from participants in order to take part to the online survey. The present study protocol has been reviewed and approved by the Ethical Review Board of the University of Campania “L. Vanvitelli” (Protocol number:0007593/i).

### Statistical analysis

Sociodemographic and clinical characteristics of the global sample have been analyzed using descriptive statistics and frequency tables, as appropriate. Differences in levels of PTG according to the different target groups (i.e., general population, healthcare workers, patients with pre-existing mental disorders, and people infected by COVID-19) were evaluated using chi-square with multiple comparisons and ANOVA with Bonferroni corrections.

In order to assess the impact of the duration of lockdown on the different dimensions of post-traumatic growth (i.e., personal strength, relating to others, new possibilities, spiritual life, and appreciation for life) multivariate linear regression models were implemented. This statistical approach has been already adopted in previous published papers based on the COMET study [54] and the categorical variable “Week” was entered in the regression models. Several sociodemographic characteristics, including gender, age, working status, having a physical comorbid condition, having a pre-existing mental disorder, civil status, level of education, satisfaction with one’s own life, and with housing conditions, adaptive and maladaptive coping strategies, having been infected by COVID-19 were entered in the models and adjusted for them.

Multiple imputation approach has been used for managing missing data. The level of statistical significance was set at *p* < 0.05 and statistical analyses were performed using the Statistical Package for Social Sciences (SPSS), version 26.0, and STATA, version 15.

## Results

The final sample consists of 20,720 participants, mainly female (71%, *N* = 14,720) and with a mean age of 40.4 ± 14.3 years (Table 1), half of the respondents were in a stable relationship and were living with a partner.

**Table 1.** Sociodemographic characteristics of the global sample (*n* = 20,720).

Age, years, mean ± SD	40.4 ± 14.3
Age groups, % (*n*)	
18–24 years old	15.2 (3,151)
25–55 years old	65.2 (13,514)
55–64 years old	14.0 (2,904)
Over 65 years old	5.6 (1,151)
Gender, *F*, % (*n*)	71 (14,720)
Living with partner, yes, % (*n*)	52.2 (10,808)
University degree, yes, % (*n*)	62 (12,844)
Employed, yes, % (*n*)	70 (14,518)
Lost job due to the pandemic, yes, % (*n*)	6.3 (1,302)
Are you practicing smart working, yes, % (*n*)	34.2 (7,089)
Spending more time on Internet, yes, % (*n*)	80.1 (16,598)
Any comorbid physical condition(s), yes, % (*n*)	14.5 (3,012)
Any mental health problem(s), yes, % (*n*)	5.5 (1,133)
Have you been infected by COVID-19, yes, % (*n*)	1.4 (296)
Have you been isolated due to COVID-19 infection, yes, % (*n*)	1.5 (316)
Have you been in contact with someone affected by COVID-19, % (*N*)	4.2 (866)

**Figure 1.**
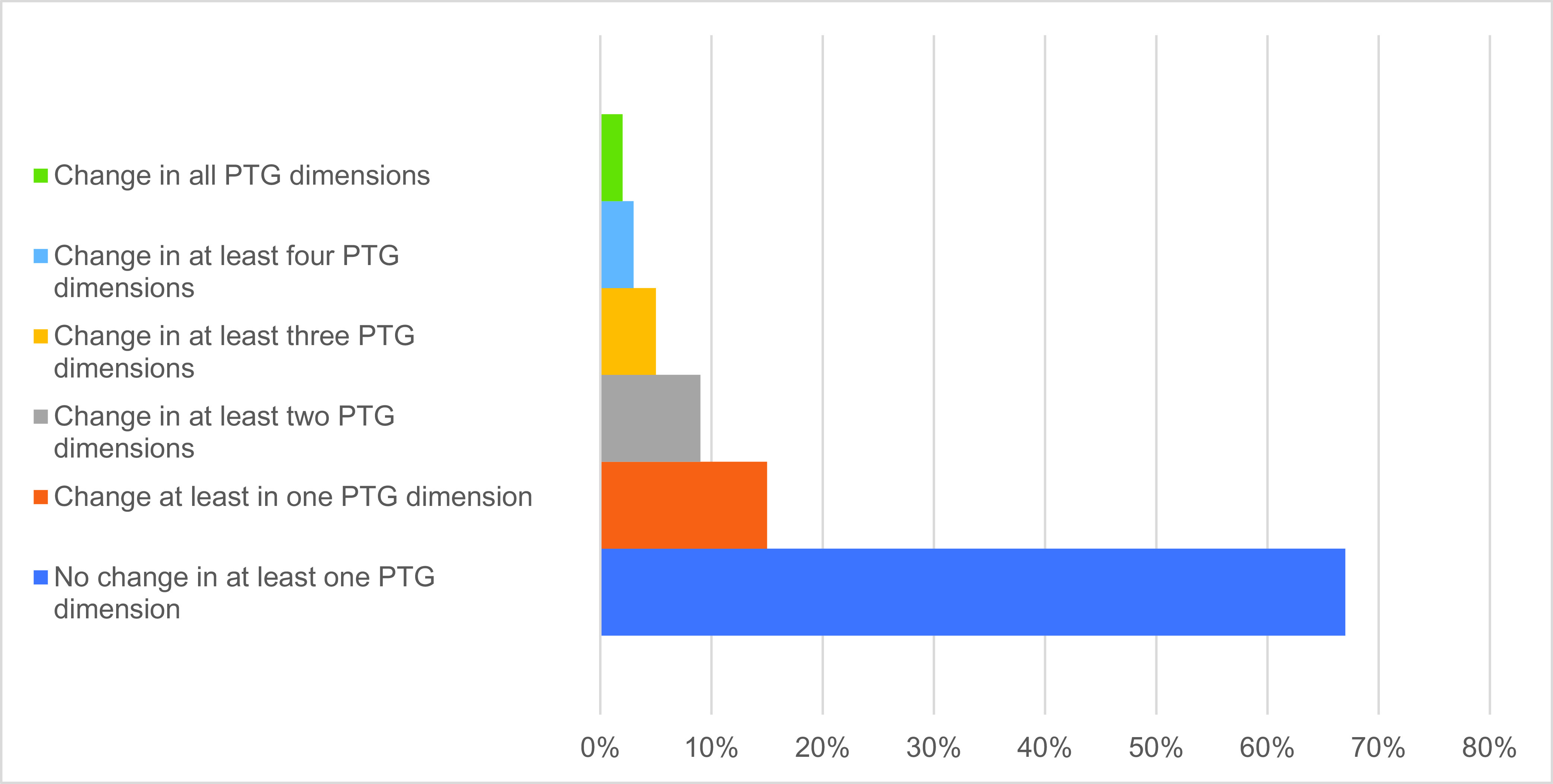
Percentage of participants with growth in at least one domain of PTG.

The majority of the sample (67%, *N* = 13,889) did not report any significant improvement in any domain of PTG (Figure 1). Only 4% of participants (*N* = 824) reported a substantial PTG (i.e., >4.0) by the overall scale score. Considering the specific dimensions of PTG, 18% (*N* = 3,739) of respondents achieved a significant post-traumatic growth in the dimensions of appreciation for life and personal strength, while only 4.8% (*N* = 1,003) of participants reported a change in spiritual life.

Participants reported the highest levels of growth in the dimension of “appreciation of life” (2.3 ± 1.4), while the lowest level was found in the “spiritual change” (1.2 ± 1.2).

Female participants reported a slightly higher level of PTG in the dimensions of personal strength (*p* < 0.002) and appreciation for life (*p* < 0.007) compared to male participants, while no significant association was found with age (Table 2). No significant differences in the levels of PTG were found among healthcare professionals, people infected by COVID-19 and patients with pre-existing mental disorders, compared to the general population (Table 3).

**Table 2.** Gender differences in levels of PTG.

		*M*	*SD*	*p*
PTGI—Relating to others	Male	1.9	1.4	0.094
	Female	1.9	1.4	
PTGI—New possibilities	Male	1.8	1.3	0.039
	Female	1.9	1.3	
PTGI—Personal strength	Male	2.1	1.5	0.002
	Female	2.2	1.5	
PTGI—Spiritual help	Male	1.1	1.2	0.310
	Female	1.2	1.2	
PTGI—Appreciation of life	Male	2.2	1.4	0.007
	Female	2.3	1.4	

Abbreviations: *M*, mean; NS, not significant; PTGI, post-traumatic growth inventory; *SD*, standard deviation; p = *p* value.

**Table 3.** Differences in the levels of PTG.

	General population		Healthcare workers		People with pre-existing mental disorder		People with COVID+		*p*
	*M*	SD	*M*	SD	*M*	SD	*M*	SD	
PTGI—Appreciation of life	2.3	1.4	2.3	1.4	2.3	1.5	2.2	1.4	NS
PTGI—Personal strength	2.2	1.5	2.2	1.5	2.1	1.6	2.1	1.5	NS
PTGI—Relating to others	1.9	1.4	1.9	1.4	1.9	1.5	1.9	1.4	NS
PTGI—New possibilities	1.8	1.3	1.8	1.3	1.8	1.4	1.8	1.3	NS
PTGI—Spiritual help	1.2	1.2	1.3	1.2	1.2	1.3	1.2	1.2	NS

Abbreviations: *M*, mean; NS, not significant; *p*, *p* value; PTGI, post-traumatic growth inventory; SD, standard deviation.

At the multivariate regression models, weighted for the propensity score, only the initial week of lockdown (between April 9 and April 15) had a negative impact on the dimension of “relating to others” of the PTG (*B* = −0.107, 95% CI = −0.181 to −0.032, *p* < 0.005), while over time no other effects were found. However, the duration of exposure to lockdown measures did not influence the other dimensions of PTG (Table 4).

**Table 4.** Predictors of levels of post-traumatic growth.

	Relating to others				New possibilities				Personal strength				Spiritual				Appreciation for life			
	*B*	Sig.	Confidence interval 95%		*B*	Sig.	Confidence interval 95%		*B*	Sig.	Confidence interval 95%		*B*	Sig.	Confidence interval 95%		*B*	Sig.	Confidence interval 95%	
			Lower bound	Upper bound			Lower bound	Upper bound			Lower bound	Upper bound			Lower bound	Upper bound			Lower bound	Upper bound
Intercept	−1.45	0.000	−1.76	−1.13	−1.33	0.000	−1.60	−1.05	−1.59	0.000	−1.92	−1.27	−1.11	0.000	−1.40	−0.903	−0.277	0.110	−0.618	0.063
Time to exposure, ref. week March 30–April 8																				
Week April 15–April 9	−0.107	0.005	−0.181	−0.032	−0.021	0.512	−0.086	0.043	−0.047	0.230	−0.124	0.030	−0.013	0.658	−0.072	0.045	−0.025	0.546	−0.105	0.056
Week April 16–April 22	−0.036	0.225	−0.093	0.022	−0.012	0.627	−0.062	0.037	0.023	0.456	−0.037	0.082	−0.015	0.515	−0.061	0.030	−0.001	0.963	−0.064	0.061
Week April 23–April 29	−0.027	0.390	−0.090	0.035	0.048	0.079	−0.006	0.102	0.052	0.112	−0.012	0.117	0.046	0.068	−0.003	0.095	0.065	0.059	−0.002	0.132
Week April 30–May 4	−0.038	0.112	−0.084	0.009	−0.007	0.725	−0.047	0.033	−0.019	0.435	−0.067	0.029	−0.012	0.504	−0.049	0.024	−0.022	0.388	−0.072	0.028
Quarantine, yes	0.027	0.702	−0.110	0.163	−0.024	0.690	−0.141	0.094	0.064	0.373	−0.077	0.205	−0.035	0.520	−0.143	0.072	−0.077	0.308	−0.224	0.071
Severely hit area	0.005	0.773	−0.031	0.042	−0.032	0.046	−0.064	−0.001	−0.021	0.285	−0.059	0.017	−0.017	0.256	−0.046	0.012	−0.023	0.265	−0.062	0.017
Gender, female ref.	−0.003	0.867	−0.041	0.034	−0.014	0.393	−0.047	0.018	−0.040	0.046	−0.079	−0.001	0.014	0.356	−0.016	0.044	−0.016	0.429	−0.057	0.024
Healthcare worker	−0.050	0.442	−0.179	0.078	−0.035	0.539	−0.146	0.076	0.034	0.621	−0.100	0.167	−0.069	0.180	−0.171	0.032	−0.090	0.204	−0.229	0.049
Being infected by COVID	0.083	0.163	−0.034	0.200	0.000	0.994	−0.101	0.100	0.140	0.024	0.019	0.261	0.007	0.882	−0.085	0.099	0.017	0.795	−0.110	0.143
Pre-existing mental disorder	−0.025	0.729	−0.163	0.114	0.049	0.421	−0.070	0.168	−0.072	0.325	−0.216	0.071	0.028	0.616	−0.081	0.137	0.119	0.118	−0.030	0.269
Pre-existing physical disorder	0.021	0.414	−0.029	0.070	−0.009	0.671	−0.052	0.033	0.015	0.556	−0.036	0.067	0.010	0.621	−0.029	0.049	0.002	0.954	−0.052	0.055
Age group, ref. over 65 years old																				
18–24 years old	−0.003	0.955	−0.097	0.092	0.005	0.905	−0.077	0.086	0.003	0.946	−0.095	0.101	0^a^	.	.	.	0.077	0.140	−0.025	0.179
25–55 years old	0.025	0.539	−0.054	0.103	0.029	0.395	−0.038	0.097	0.058	0.161	−0.023	0.140	0.061	0.108	−0.013	0.136	0.068	0.114	−0.017	0.153
55–64 years old	0.118	0.007	0.033	0.204	0.056	0.134	−0.017	0.130	0.122	0.007	0.033	0.211	0.065	0.040	0.003	0.127	0.130	0.006	0.038	0.223
Resilience level	0.025	0.000	0.023	0.027	0.037	0.000	0.035	0.038	0.047	0.000	0.045	0.049	0^a^	.	.	.	0.020	0.000	0.018	0.022
Level of education	0.027	0.001	0.011	0.042	0.007	0.287	−0.006	0.021	0.012	0.149	−0.004	0.028	0.020	0.000	0.019	0.022	0.007	0.448	−0.010	0.023
Satisfaction	−0.006	0.163	−0.014	0.002	−0.002	0.596	−0.009	0.005	−0.003	0.454	−0.012	0.005	0.004	0.542	−0.008	0.016	−0.009	0.054	−0.018	0.000
Satisfaction—cohabitants	−0.003	0.453	−0.011	0.005	−0.002	0.538	−0.009	0.005	−0.006	0.137	−0.015	0.002	−0.006	0.084	−0.013	0.001	−0.005	0.236	−0.014	0.003
Satisfaction—living conditions	0.000	0.910	−0.008	0.009	0.001	0.682	−0.006	0.009	0.001	0.759	−0.007	0.010	−0.002	0.611	−0.008	0.005	0.000	0.951	−0.009	0.009
Support—family	0.013	0.000	0.010	0.017	−0.003	0.016	−0.006	−0.001	0.005	0.002	0.002	0.009	0.003	0.328	−0.003	0.010	0.004	0.015	0.001	0.008
Support—friends	0.035	0.000	0.031	0.038	0.007	0.000	0.004	0.010	0.007	0.000	0.004	0.011	−0.002	0.143	−0.004	0.001	0.005	0.005	0.001	0.009
Support—others	0.007	0.000	0.004	0.010	0.003	0.068	0.000	0.005	0.012	0.000	0.008	0.015	−0.001	0.624	−0.003	0.002	0.010	0.000	0.006	0.013
COPE active coping	0.006	0.684	−0.022	0.033	0.045	0.000	0.022	0.069	0.043	0.003	0.015	0.072	0.006	0.000	0.004	0.009	0.035	0.022	0.005	0.065
COPE denial	0.217	0.000	0.190	0.244	0.118	0.000	0.095	0.142	0.208	0.000	0.180	0.237	0.013	0.245	−0.009	0.035	0.248	0.000	0.219	0.278
COPE substance abuse	−0.101	0.000	−0.133	−0.069	0.003	0.842	−0.025	0.031	−0.120	0.000	−0.153	−0.087	0.144	0.000	0.122	0.165	−0.015	0.395	−0.050	0.020
COPE emotional support	0.140	0.000	0.108	0.172	0.055	0.000	0.028	0.083	0.071	0.000	0.038	0.104	−0.030	0.019	−0.056	−0.005	0.048	0.007	0.013	0.082
COPE practical support	0.186	0.000	0.153	0.218	0.022	0.120	−0.006	0.050	0.093	0.000	0.060	0.127	0.010	0.422	−0.015	0.035	0.066	0.000	0.031	0.102
COPE emotional disengagement	−0.063	0.000	−0.093	−0.034	−0.064	0.000	−0.089	−0.038	−0.102	0.000	−0.133	−0.071	0.067	0.000	0.042	0.093	−0.037	0.024	−0.069	−0.005
COPE venting	−0.043	0.002	−0.070	−0.016	0.071	0.000	0.047	0.094	0.034	0.017	0.006	0.063	−0.027	0.025	−0.050	−0.003	0.066	0.000	0.037	0.096
COPE reframing	0.208	0.000	0.182	0.233	0.142	0.000	0.120	0.164	0.247	0.000	0.221	0.274	0.030	0.007	0.008	0.051	0.177	0.000	0.149	0.204
COPE planning	−0.092	0.000	−0.122	−0.061	−0.002	0.897	−0.028	0.024	−0.017	0.282	−0.049	0.014	0.088	0.000	0.068	0.108	−0.029	0.086	−0.061	0.004
COPE humor	−0.123	0.000	−0.148	−0.098	−0.091	0.000	−0.112	−0.070	−0.150	0.000	−0.175	−0.124	0.002	0.859	−0.022	0.026	−0.170	0.000	−0.197	−0.143
COPE acceptance	−0.030	0.037	−0.057	−0.002	−0.029	0.018	−0.053	−0.005	−0.025	0.095	−0.053	0.004	−0.093	0.000	−0.113	−0.074	−0.023	0.137	−0.053	0.007
COPE religion	0.217	0.000	0.199	0.235	0.520	0.000	0.504	0.535	0.224	0.000	0.205	0.243	−0.105	0.000	−0.127	−0.083	0.137	0.000	0.117	0.157
COPE self-blame	−0.047	0.000	−0.073	−0.022	0.032	0.004	0.010	0.054	0.009	0.506	−0.017	0.035	0.632	0.000	0.618	0.646	0.019	0.164	−0.008	0.046
COPE self-distraction	0.152	0.000	0.129	0.174	0.101	0.000	0.081	0.120	0.178	0.000	0.154	0.202	0.049	0.000	0.029	0.069	0.211	0.000	0.187	0.236
Civil status, divorced	−0.146	0.060	−0.298	0.006	−0.105	0.115	−0.237	0.026	−0.228	0.005	−0.386	−0.070	0.092	0.000	0.073	0.110	−0.048	0.568	−0.212	0.116
Single	−0.060	0.417	−0.206	0.085	−0.030	0.639	−0.155	0.095	−0.153	0.047	−0.303	−0.002	−0.059	0.333	−0.179	0.061	−0.036	0.652	−0.193	0.121
With partner/married	−0.106	0.143	−0.248	0.036	−0.070	0.264	−0.192	0.053	−0.194	0.010	−0.341	−0.047	−0.034	0.562	−0.148	0.081	−0.056	0.472	−0.209	0.097
Widow	0	.	.	.	0	.	.	.	0	.	.	.	−0.085	0.137	−0.196	0.027	0	.	.	.
	*R* ^2^ = 0.257; *R* ^2^ adjusted = 0.255				*R* ^2^ = 0.348, *R* ^2^ adjusted = 0.346				*R* ^2^ = 0.282; *R* ^2^ adjusted = 0.281				*R* ^2^ = 0.367, *R* ^2^ adjusted = 0.366				*R* ^2^ = 0.132; *R* ^2^ adjusted = 0.130			

Abbreviations: *B* = beta coefficient; Model statistics: *R* ^2^ = *R* ^2^ adjusted. Sig = significance.

Factors significantly associated with the increase in the levels of PTG include the levels of resilience, with a *B* coefficient ranging from .025 (95% CI = 0.023 to 0.027) for “relating to others” (*p* < 0.000) to *B* = 0.047 (95%CI = 0.045 to 0.049) for “personal strength” (*p* < 0.000), the perceived support from family members and friends and the level of education. Furthermore, adaptive coping strategies, such as emotional support (*B* = 0.140, 95% CI = 0.108 to 0.172, dimension “relating to others”; *B* = 0.055, 95% CI = 0.028 to 0.083, dimension “new possibilities”; *B* = 0.071, 95% CI = 0.038 to 0.104; *B* = 0.048, 95% CI = 0.013 to 0.082, dimension “appreciation for life”), reframing (0.208, 95% CI = 0.182 to 0.233) and practical support (*B* = 0.186, 95% CI = 0.153 to 0.218) were significant predictors of several dimensions of PTG, including relating to others, new possibilities and appreciation for life. On the other hand, maladaptive coping strategies, including self-blame (*B* = −0.047, 95% CI = −0.073 to −0.022) and venting (*B* = −0.043, 95% CI = −0.070 to −0.016) were associated with a reduction of many dimensions of post-traumatic growth.

Living in one of the most severely hit areas of the pandemic was a negative predictor only for the “New possibilities” (*B* = −0.032, 95% CI = −0.064 to −0.001), but not for the other dimensions of PTG. Having a pre-existing mental or physical disorder, having being infected by COVID-19, being a healthcare worker did not have any impact on the several dimensions of post-traumatic growth.

Finally, in the different age groups, the probability of having higher levels of post-traumatic growth was found in people aged 55–64 years old, both for the dimension of relating to others (*B* = 0.118, 95% CI = 0.033 to 0.204) as well as for the dimension of personal strength (*B* = 0.122, 95% CI = 0.033 to 0.211).

## Discussion

This study was conducted to investigate the levels of post-traumatic growth during the first wave of COVID-19 related lockdown in the general population. During the initial phase of the national emergency for the pandemic, Italy was one of the most severely hit areas in Europe, and strict containment measures were issued by the Italian government in order to limit the spread of the virus and its morbidity and mortality rate, since no vaccinations were available [91]. This survey was promoted and disseminated in the Italian general population during the weeks of the first lockdown, a period of uncertainty, fears for the future and exceptional changes in the daily routine. All these sociocultural factors have contributed to feature the pandemic as a new type of traumatic stressor, which could have an impact on the mental health of the general population. Although several papers have reported increasing levels of anxiety, depressive and stress symptoms in the Italian general population [54], as well as the presence of sleep disorders and of suicidal ideation, a few data are available on the possible positive consequences of the pandemic on the general population. Some studies have found that growth and distress are at opposite ends of the same continuum, from which a negative association was found [92]. Alternatively, growth has been thought to positively coexist with distress, with some authors stating that “the higher the distress, the better the growth” [93]. In the present study, we found that respondents did not report high levels of post-traumatic growth, with only 15% reporting a significant growth at least in one dimension. This data is in line with those found in Hong-Kong, where post-traumatic growth was found in less than 20% of the general population [94,95]. Other studies carried out in China reported levels of post-traumatic growth of up to 50% in at least one domain of PTG. These differences could be due to the divergence in social contexts among countries, in terms of social cohesion, acceptance and satisfaction with the governmental measures for containing the pandemic and the perception of collective identity [96,97]. Therefore, it is of extreme interest to understand the possible impact of these necessary and unavoidable containment measures on the mental health of the general population, in order to develop appropriate supportive and preventive interventions to mitigate the long-term negative effects of the pandemic on mental health.

Regarding the several PTG dimensions, we found that scores of “appreciation of life” were the greatest, while “spiritual change” was the lowest. These results are in line with those reported by Prati and Pietrantoni [98], confirming that our findings can be considered representative of the Italian general population.

Another interesting finding is that higher levels of post-traumatic growth during the initial phase of the pandemic were found in female participants. Previous studies carried out during other natural emergencies have found a gender difference in the levels of post-traumatic growth [99]. Although little research has examined the underlying processes for such gender differences in PTG, the role of some cognitive styles, such as rumination, has been proposed [99,100]. In particular, the tendency to ruminate on constructive issues, such as an increased awareness of personal strengths or an appreciation of the importance of social connections, has been suggested as the mechanism leading to the greater reports of PTG [101]. In different groups of traumatized people, such as bereaved parents or women at a high risk for breast cancer, the use of reflective rumination was associated with high levels of post traumatic growth [102–104].

Another potential mediator while processing traumatic events is the type of coping strategies adopted. In fact, we found that using adaptive coping strategies, such as planning, practical support and reframing, predicted higher levels of post-traumatic growth. This finding is in line with previous COVID-related data [54,105] but also with other studies carried out on factors moderating the impact of traumatic events [101,106,107]. PTG may be conceptualized as a cognitive adaptive process among those who experience traumatic stress in response to a disaster, in terms of a positive reinterpretation and positive reframing of the negative experience. However, the use of adaptive coping strategies can sustain and booster this process and it is therefore essential to promote the dissemination of psychosocial interventions aiming to teach and improve adaptive coping strategies in the general population.

Contrary to what we expected, we did not find a significant effect of the weeks of lockdown on the levels of post-traumatic growth, except for the dimension of “searching new possibilities.” This finding is particularly striking if we consider that the levels of stress and of psychiatric symptoms tended to increase over time [54]; it may be that PTG is not related to the duration of the traumatic event, but it is related to the nature of the trauma and to the personality traits and characteristics of the individual [108]. Of course, this interpretation deserves more studies. Furthermore, patients with pre-existing severe mental disorders did not show significantly lower levels of PTG, compared to the general population. This was an unexpected finding, which should be due to the ability, skills and personal resources of patients to adapt to the “new” life routine posed by the pandemic. Moreover, a possible time-lead effect should explain this finding, being the levels of PTG quite high at the initial phase of the pandemic, and it should be reduced over the following months.

The present study has some limitations, which are hereby acknowledged. First, the online snowball sampling methodology may have led to a selection bias, with only those interested in the psychological consequences of the pandemic willing to participate [109]. Second, the cross-sectional design of the survey prevents us to delineate any causal relationship between the selected variables. Finally, several variables, such as social cohesion, national identity and interpersonal trust, personality traits and cognitive styles should have had an impact on the levels of post-traumatic growth [108,110].

## Conclusions

The assessment of the levels of post-traumatic growth in the general population during the initial phase of the national health emergency is of great importance for the development of ad hoc supportive and preventive psychosocial interventions [111–114]. It has been repeatedly stated that the pandemic will have longstanding, and far-reaching, consequences on global mental health and wellbeing to the whole population, regardless of age and gender [115–118]. From a public health perspective, the identification of protective factors is crucial for developing ad hoc tailored interventions and for preventing the development of full-blown mental disorders in large scale [119–122]. From a clinical practice perspective, the promotion of supportive interventions aiming to improve the levels of resilience, the adaptive coping strategies and the levels of post-traumatic growth should be prioritized in order to mitigate the detrimental effects of the pandemic.

## Data Availability

The dataset is not available for sharing.
